# Identification of Coagulation and Fibrinolysis-Associated Biomarkers With Implications for Preeclampsia

**DOI:** 10.1155/genr/6637484

**Published:** 2025-06-23

**Authors:** Yujie Liu, Tingting Chen, Cuifang Fan

**Affiliations:** Department of Obstetrics and Gynecology, Renmin Hospital of Wuhan University, Wuhan, Hubei, China

**Keywords:** coagulation, fibrinolysis, GEO, novel biomarkers, preeclampsia, WGCNA

## Abstract

**Background:** Coagulation system abnormalities contribute to clinical manifestations in preeclampsia (PE), but the mechanisms of coagulation and fibrinolysis in PE are unclear.

**Methods:** We utilized the Gene Expression Omnibus (GEO) database to obtain the GSE10588 training set and GSE54618 validation set. From GeneCards, we extracted 514 coagulation and fibrinolysis-related genes (CFRGs). Differential expression analysis identified 1521 DEGs in the GSE10588 training set. WGCNA revealed the salmon module (778 genes) as the key module. LASSO and SVM-RFE methods identified four biomarkers (CYP19A1, C1QBP, GHR, and PSMA3) for a diagnostic model. GSEA was performed on the biomarkers. Immune cell infiltration and therapeutic agents for the biomarkers were analyzed. A circRNA-miRNA-mRNA network was constructed.

**Results:** The salmon module showed the highest correlation with PE and normal samples. The diagnostic model comprised CYP19A1, C1QBP, GHR, and PSMA3. Immune cell analysis revealed significant differences, including type 2 T helper cells and regulatory T cells. C1QBP correlated positively with effector memory CD4 T cells, while PSMA3 had a negative correlation with CD56dim natural killer cells. Sixty-one potential therapeutic agents were predicted, as well as n circRNA-miRNA-mRNA network composed of 73 nodes and 88 edges.

**Conclusion:** Our bioinformatic analysis resulted in a diagnostic model (CYP19A1, C1QBP, GHR, and PSMA3) for PE related to coagulation and fibrinolysis. We also conducted immune microenvironment and drug sensitivity analyses, providing insights into PE diagnosis and treatment.

## 1. Introduction

Preeclampsia is a clinical condition characterized by the onset of hypertension and proteinuria occurring after the 20th week of gestation, accompanied by symptoms such as headache, visual disturbances, nausea, vomiting, and upper abdominal discomfort [1]. It represents a significant obstetric concern. PE affects 3%–8% of pregnancies in the United States and 1.5%–16.7% globally, contributing to 60,000 maternal deaths and 500,000 preterm births annually worldwide [2]. Statistics reveal that PE is the second leading cause of maternal mortality [3]. PE can lead to various organ disorders, including proteinuria, acute kidney injury, liver dysfunction, hemolysis, low platelet count, seizures (eclampsia), stroke, and death [2]. Currently, the only definitive treatment for PE is the delivery of the placenta and fetus, often resulting in iatrogenic preterm birth [4]. The precise mechanisms underlying the occurrence and development of PE remain unclear [5]. Therefore, it is essential to explore biomarkers associated with PE to establish a theoretical foundation for understanding the mechanisms and clinical diagnosis of this condition.

The coagulation and immune systems form an interconnected network that efficiently responds to injury and combats pathogen invasion [6]. Research has indicated significant alterations in the coagulation system of PE patients during the antepartum and even postpartum periods. These changes include increased levels of coagulation factors, thrombin, platelet activation, decreased levels of anticoagulant proteins, and impaired fibrinolysis, primarily involving alterations in PAI-1 and PAI-2 levels. In PE, particularly when accompanied by organ dysfunction such as renal impairment, elevated levels of coagulation factors may be implicated [7]. Pathological hypercoagulability and an increased propensity for thrombus formation due to abnormalities in the coagulation system may contribute to the various clinical manifestations of PE [8]. Currently, antiplatelet and anticoagulant medications remain crucial in the prevention and treatment of PE [9]. Therefore, understanding the role of coagulation and fibrinolysis-related genes (CFRGs) in the development of PE can provide new insights for further research and therapeutic strategies targeting the mechanisms of PE.

We downloaded a dataset related to PE from the GEO database. A total of 514 CFRGs were extracted from the GeneCards database. Utilizing techniques such as differential expression analysis, weighted gene co-expression network analysis (WGCNA) [10], Lasso regression analysis [11], and SVM-RFEAPE, we successfully identified a comprehensive panel of biomarkers linked to coagulation and fibrinolysis. To establish a diagnostic model utilizing these biomarkers, gene set enrichment analysis (GSEA) was performed. Furthermore, we analyzed immune cell infiltration, predicted targeted therapeutic drugs for the biomarkers, and constructed a circRNA-miRNA-mRNA network. Investigating the mechanisms of coagulation and fibrinolysis-related biomarkers in the development of PE holds significant importance for the diagnosis and treatment of the disease and related research.

## 2. Materials and Methods

### 2.1. Sources of Data

The GSE10588 training set, consisting of 17 PE samples and 26 normal samples, sourced from placental tissue, was obtained from the Gene Expression Omnibus (GEO) database (https://www.ncbi.nlm.nih.gov/gds). The platform used for this set was the ABI Human Genome Survey Microarray Version 2. Additionally, the GSE54618 validation set, comprising 5 PE samples and 12 normal samples, also derived from placental tissue, was obtained from the same GEO database. The platform utilized for this set was the Illumina HumanHT-12 V4.0 expression beadchip. To identify relevant genes, we extracted 514 CFRGs from the GeneCards database (https://www.genecards.org/), based on a previously published article.

### 2.2. Differential Expression Analysis and WGCNA

Differentially expressed genes (DEGs) between PE and normal samples in GSE10588 training set were sifted out by limma package (version 3.54.1) setting adj. *p* < 0.05 and |log_2_FC)| > 0.5. Then, we considered PE and normal as the traits for WGCNA to analyse all genes in the GSE10588 training set. Outlier detection and elimination were implemented, and the module with the highest correlation with PE and normal was screened out as key module by constructing co-expression network. Furthermore, DEGs, genes in the key module, and CFRGs were intersected to yield intersected genes, and they were analyzed for gene ontology (GO) and kyoto encyclopedia of genes and genomes (KEGG) functional enrichment by clusterProfiler package (version 4.6.2) [12] setting *p* < 0.05.

### 2.3. Construction of a Diagnostic Model

First, least absolute shrinkage and selection operator (LASSO) regression analysis and support vector machine-recursive feature elimination (SVM-RFE) were respectively implemented on intersected genes by glmnet package (version 4.1-4) [13] (setting family to binomial) and e1071 package (version 1.7-13) [14] to acquire the corresponding genes. After that, the genes gained from above two algorithms were intersected via online venn tool (https://jvenn.toulouse.inra.fr/app/example.html) to identify biomarkers. Finally, we constructed a diagnostic model based on the biomarkers utilizing the glm function. In order to evaluate the diagnostic performance of the diagnostic model for PE, we extracted the expression values of biomarkers in the GSE10588 training set, and plotted receiver operating characteristic (ROC) curve with the grouping information of samples in the dataset. We also validated the evaluation results by the same methods in GSE54618 validation set.

### 2.4. Functional Enrichment Analysis of Biomarkers

The background gene set “c2.cp.kegg.v7.4.1.symbols.gmt” was obtained from the Molecular Signatures Database (MSigDB) (https://www.gsea-msigdb.org/gsea/msigdb/index.jsp). Subsequently, we computed the Pearson correlation coefficients between the identified biomarkers and all genes within the GSE10588 training set. The correlation coefficients were then ranked in descending order. Finally, GSEA using GO and KEGG was conducted on the biomarkers using the clusterProfiler package (version 4.6.2). The significance threshold was set at adj.*p* < 0.05.

### 2.5. Immune Cell Infiltration Analysis

Immune cell infiltration analysis was conducted on PE and normal samples in the GSE10588 training set utilizing single-sample GSEA (ssGSEA) algorithm to yield immune cell scores. Next, we compared the differences of these scores between PE and normal samples, and the immune cells with significant differences were extracted for Spearman correlation analysis with biomarkers.

### 2.6. Tissue-Specific Expression and Analysis of Related Genes for Biomarkers

Tissue-specific expressions of biomarkers in various systems and organs were analyzed via BioGPS website (https://biogps.org). Afterwards, we utilized GeneMANIA database (https://genemania.org/) to sift out the genes associated with biomarkers, and their interactions were analyzed.

### 2.7. Construction of the Biomarker-Drug and Long Noncoding RNAs (lncRNAs)-miRNA-mRNA Networks

We predicted potential therapeutic agents targeting biomarkers through DrugBank database (https://go.drugbank.com), and constructed a biomarker-drug network based on them. Subsequently, the miRNAs for biomarkers were predicted via miRnet database (https://www.mirnet.ca/) (selected placental tissues), and lncRNAs were predicted based on the targeted miRNAs through Starbase database (https://starbase.sysu.edu.cn/starbase2/) (filtered with strong stringency).

### 2.8. Quantitative Reverse Transcription Polymerase Chain Reaction (qRT-PCR) Analysis

The experimental workflow involved the isolation of RNA, followed by qRT-PCR analysis. To validate the obtained results, RNA was extracted from human placental tissue and subjected to qRT-PCR analysis. Total RNA was extracted using RNAiso Plus (Trizol) reagent, and its concentration was determined using the NanoDrop 2000 spectrophotometer. The TSK301 reverse transcription system kit was employed for the RT reaction and subsequent qRT PCR, as per the manufacturer's instructions. For qRT-PCR analysis, SYBR Green RT-qPCR Master Mix (Servicebio, Wuhan, China) was used. Primers were designed and synthesized by Wuhan Servicebio Co., Ltd. The amplification protocol consisted of 40 cycles, with denaturation at 95°C for 10 s, annealing at 60°C for 30 s, and extension at 60°C for 30 s. GAPDH was used as the internal reference gene to normalize the expression levels of the target genes. Below is the list of primer sequences for each signature:

• CYP19A1: ACATCTGGACAGGTTGGAGGAG (sense primer)
• CYP19A1: CCACGATAGCACTTTCGTCCA (antisense primer)
• C1QBP: ACTGGGCCTTATATGACCACCT (sense primer)
• C1QBP: CATCTGTCTGCTCTACTGGCTCTT (antisense primer)
• GHR: ATGCCACTGGACAGATGAGGTT (sense primer)
• GHR: GGCAATGGGTGGATCTGGTT (antisense primer)
• PSMA3: CCCATCAGGTGTTTCATACGGT (sense primer)
• PSMA3: ATCACGGCAGGTCATTTCTTTC (antisense primer)
• GAPDH: GGAAGCTTGTCATCAATGGAAATC (sense primer)
• GAPDH: TGATGACCCTTTTGGCTCCC (antisense primer)

### 2.9. Statistics

All statistical analyses were conducted using R software (version 4.1.3) and GraphPad Prism 9.5. Group comparisons for categorical variables were assessed using Fisher's exact test, while comparisons for continuous variables were evaluated using the *t*-test assuming equal variances, unless explicitly stated otherwise. To assess the diagnostic accuracy of gene expression levels in predicting PE, ROC curve analysis and calculation of the area under the curve (AUC) values were performed. Two-tailed *t*-tests were employed to compare gene expression or scores between two groups. A *p* value less than 0.05 was considered statistically significant.

## 3. Results

### 3.1. Identification of DEGs and Key Module

**Figure 1:**
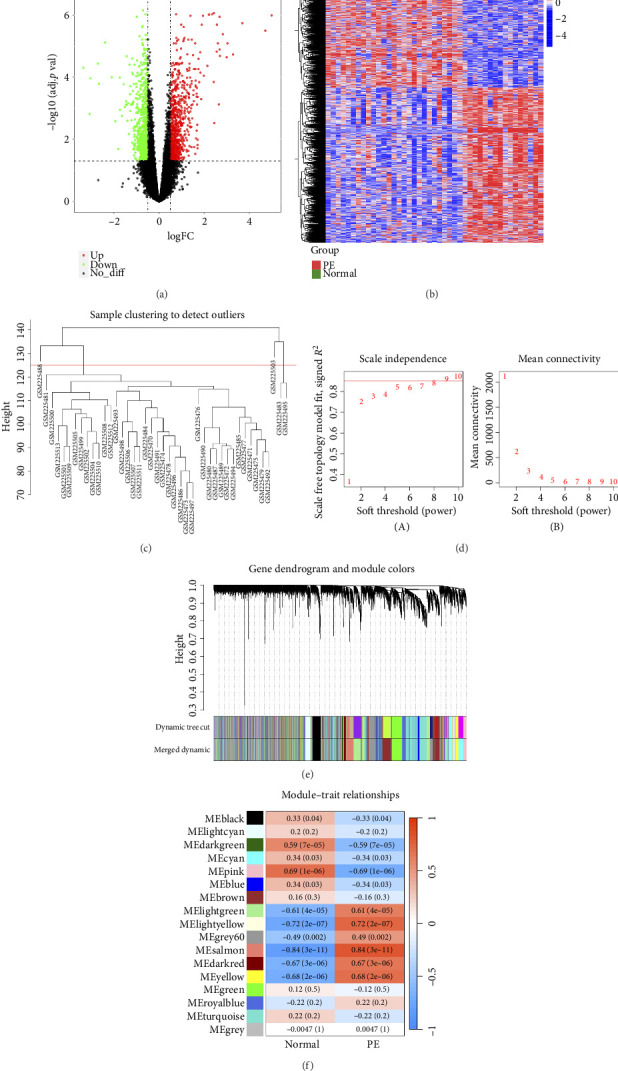
Visualization of differentially expressed genes (DEGs) and construction of WGCNA co-expression modules and selection of hub modules. (a) The volcano plot presents the DEGs identified based on the threshold (adjusted *p*-value < 0.05 and |logFC| > 1). (b) The heatmap displays the expression of the top 10 upregulated and downregulated genes, ordered by adjusted *p*-value. (c) Dendrogram of module eigengenes and heatmap of eigengene network. (d) Scale-free fit index for soft-thresholding powers. (A): relationship between soft-threshold and scale-free R^2^. (B): relationship between soft-threshold and mean connectivity. (e) Dendrogram of differentially expressed genes (DEGs) clustered in the training dataset. (f) Heatmap displaying the correlation between module eigengenes and preeclampsia (PE).

There were 1521 DEGs between PE and normal samples (Figures 1(a) and 1(b)). From Figure 1(c), we could see that there were 4 outlier samples (GSM225488, GSM225503, GSM225483, and GSM225495) in the GSE10588 training set, so they were eliminated. According to the position of red line in Figure 1(d), the soft threshold was determined to be 9. At this point, the vertical coordinate *R*^2^ was at 0.9, and mean value of adjacency function was gradually closed to 0, which indicated that the network approached the scale-free distribution and showed a flat trend. Besides, we finally gained 16 gene modules (excluded the un-categorized genes in grey module) after constructing the co-expression matrix (Figure 1(e)). Salmon module (contained 778 genes) had the highest correlation with PE and normal, therefore we treated it as the key module (|cor| = 0.84, *p*=3e − 11) (Figure 1(f)).

### 3.2. Functional Pathways Involved in Intersected Genes

**Figure 2:**
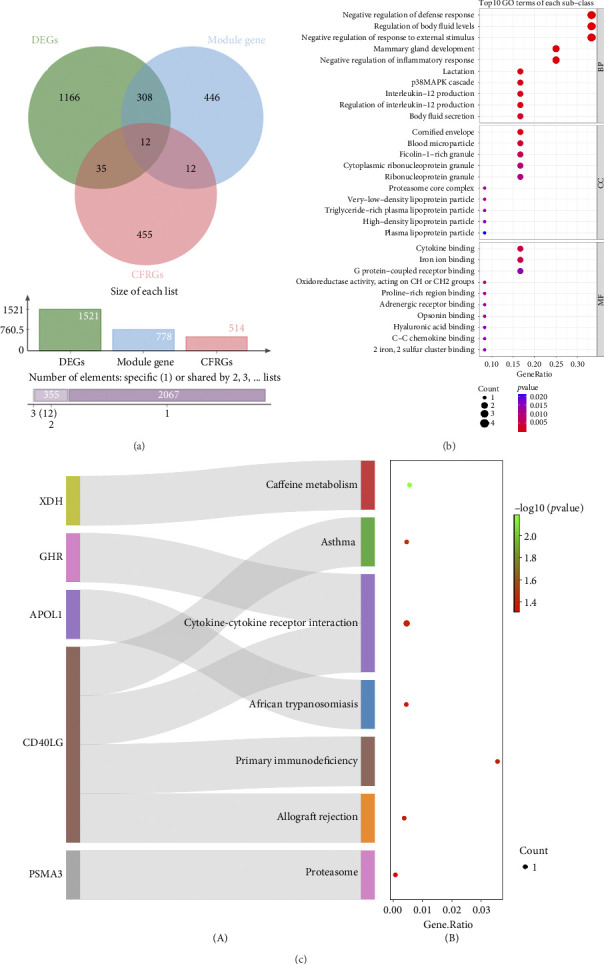
Screening of hub genes and candidate gene enrichment analysis. (a) Venn diagram illustrating differentially expressed genes DEGs, models the block genes and CFRGs were intersected to obtain 12 differentially related genes (CFR-DEGs) related to coagulation and fibrinolysis (b) Gene ontology enrichment analysis of the 12 hub genes in molecular functions. The color gradient from green to red represents the increasing significance of enrichment. The size of the dot represents the number of different genes included in the corresponding pathway. (c) A Sankey diagram was used to visualize the KEGG enrichment analysis of CFR-DEGs. The (A) side shows the gene-pathway associations, while the (B) side represents the proportion of genes in each entry relative to all genes. The color gradient from red to green indicates the confidence level of the results, with higher confidence indicated by greener shades. The results are sorted for clarity.

The DEGs, genes in the key module, and CFRGs were intersected to yield 12 intersected genes (Figure 2(a)). Meanwhile, intersected genes were enriched to 389 GO entries, including regulation of body fluid levels, blood microparticle, proteasome core complex, cytokine binding, iron ion binding, etc (Figure 2(b)). Intersected genes were also involved in 7 KEGG pathways, such as caffeine metabolism, asthma, cytokine-cytokine receptor interaction, primary immunodeficiency, and proteasome (Figure 2(c)).

### 3.3. Evaluation of the Diagnostic Model

**Figure 3:**
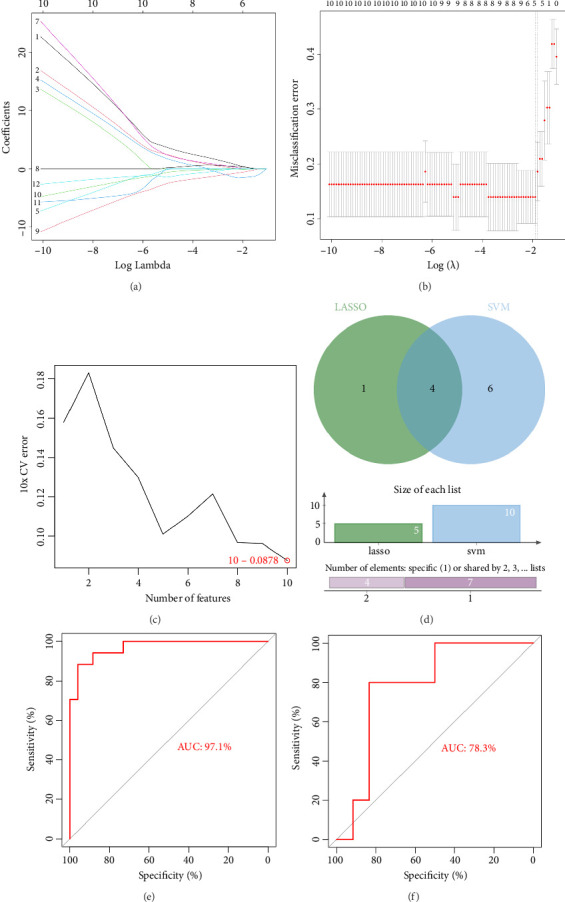
Lasso regression analysis (a) each curve depicts the trajectory of the coefficient for each independent variable. The *y*-axis represents the coefficient value, while the *x*-axis represents the number of non-zero coefficients in the model at a given time. (b) The LASSO plot shows the relationship between log (lambda) and cross-validation error. The red dots represent mean squared error and its variation, with smaller values indicating better model fit. The number above the plot indicates the remaining independent variables in the model. (c) Support vector machine model error rate (d) venn diagram of hub genes (e) logistic regression model ROC curve training set GSE10588 (f) logistic regression model ROC curve validation set GSE54618.

Five and 10 genes were identified using LASSO and SVM-RFE methods individually (Figures 3(a) and 3(b)). By intersecting the gene sets obtained from both machine learning approaches, four biomarkers were identified, namely, CYP19A1, C1QBP, GHR, and PSMA3 (Figure 3(c)). Notably, the diagnostic model demonstrated favorable performance for PE, as evidenced by the AUC values exceeding 0.75 in both the training and validation datasets (Figure 3(d)).

### 3.4. Functional Annotation Analysis of Hub Genes

**Figure 4:**
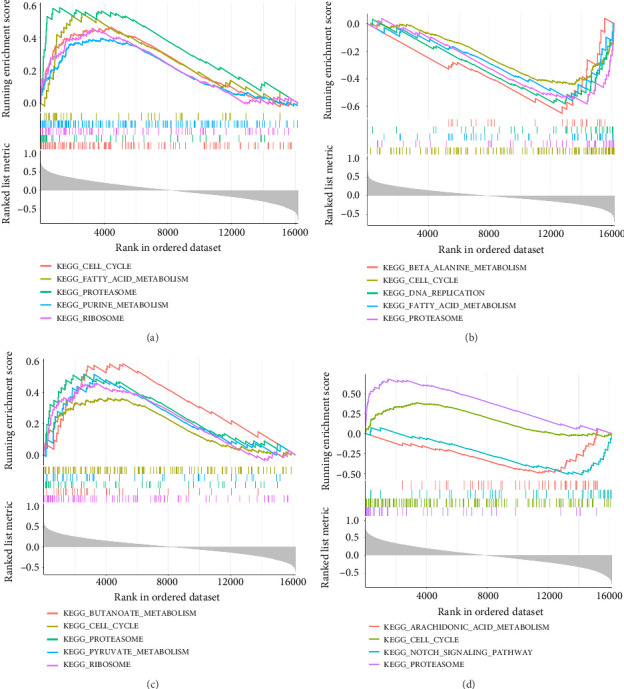
GSEA enrichment analysis: (a) significantly enriched pathways for C1QBP, (b) significantly enriched pathways for CYP19A1, (c) significantly enriched pathways for GHR, (d) significantly enriched pathways for PSMA3.

Hub genes were found to have shared involvement in cell cycle and proteasome pathways (Figure 4). Additionally, C1QBP exhibited enrichment in fatty acid metabolism, purine metabolism, ribosome, and other pathways. Similarly, CYP19A1 was associated with beta alanine metabolism, DNA replication, and other pathways. GHR showed associations with butanoate metabolism, pyruvate metabolism, ribosome, and other pathways. PSMA3 demonstrated enrichment in arachidonic acid metabolism and the notch signaling pathway.

### 3.5. Immune Microenvironment Analysis

**Figure 5:**
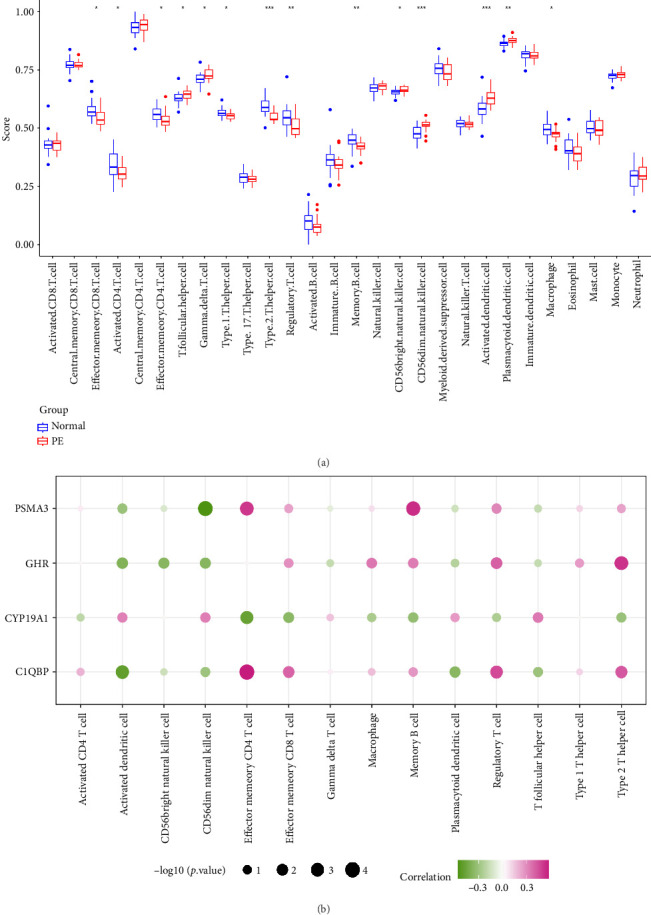
Immune cell infiltration: (a) boxplot of immune cell infiltration in PE group and normal group and (b) correlation heat map of differential immune cells and biomarkers.

Significant differences in the scores of 14 immune cells were observed between PE and normal samples, including type 2 T helper cells, regulatory T cells, and activated dendritic cells (Figure 5(a)). The correlation heat map revealed the presence of a robust positive correlation between C1QBP and effector memory CD4 T cells (*r* = 0.58, *p*=6.38e − 05), while PSMA3 exhibited the strongest negative correlation with CD56dim natural killer cells (*r* = −0.56, *p*=0.0001) (Figure 5(b)).

### 3.6. Tissue-Specific Expression and Related Genes of Core Genes

**Figure 6:**
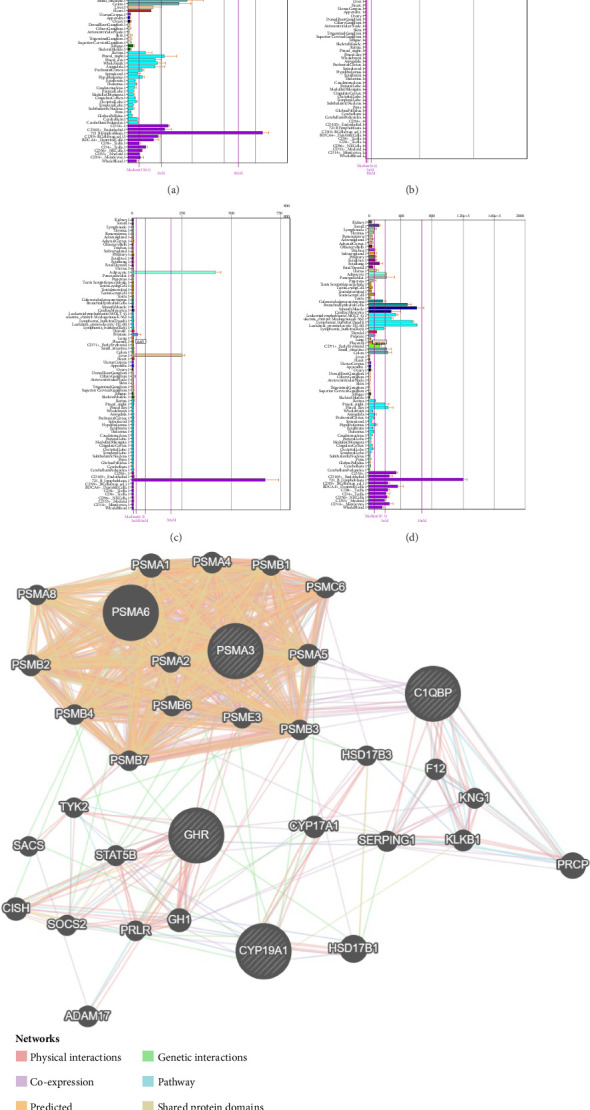
Tissue-specific expression and interaction network of biomarkers: (a) tissue-specific expression of C1QBP, (b) tissue-specific expression of CYP19A1, (c) tissue-specific expression of GHR, (d) tissue-specific expression of PSMA3, (e) related genes and interaction network diagram of biomarkers.

In the tissue-specific expression analysis of hub genes, it was observed that CYP19A1 exhibited significantly higher expression in the placenta compared to other systems and organs. Conversely, C1QBP, GHR, and PSMA3 demonstrated the highest expression levels in 721 B lymphoblasts (Figure 6(a)). Ultimately, a total of 30 core genes were identified and formed an interconnected network, which included interactions such as GHR-TYK2, C1QBP-PRCP, and CYP19A1-PRLR (Figure 6(b)).

### 3.7. The Hub Genes-Drug and lncRNA-miRNA-mRNA Networks

**Figure 7:**
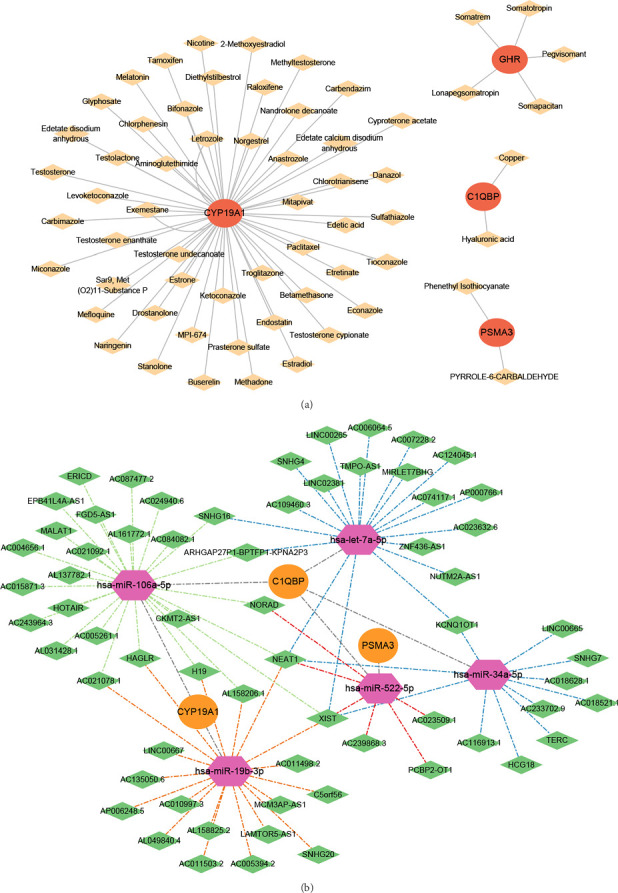
(a) Drug prediction of hub genes, the orange nodes in the figure are biomarkers, and the yellow nodes are predicted drugs. (b) ceRNA network, the orange nodes in the figure are mRNAs (hub genes), the red nodes are miRNAs, and the green nodes are circRNAs.

A total of 61 potential therapeutic agents of hub gens were predicted, which formed a hub genes-drug network containing CYP19A1-Tamoxifen, GHR-Pegvisomant, hyaluronic acid (HA)-C1QBP, and CYP19A1-Paclitaxel and other relationship pairs (Figure 7(a)). Moreover, the 73 nodes and 88 edges constructed the lncRNA-miRNA-mRNA network, which included LINC02381-hsa-let-7a-5p-C1QBP, MCM3AP-AS1-hsa-miR-19b-3p-CYP19A1, HOTAIR-hsa-miR-106a-5p-C1QBP, etc. (Figure 7(b)).

### 3.8. Validation of Hub Genes in mRNA Expression Levels

**Figure 8:**
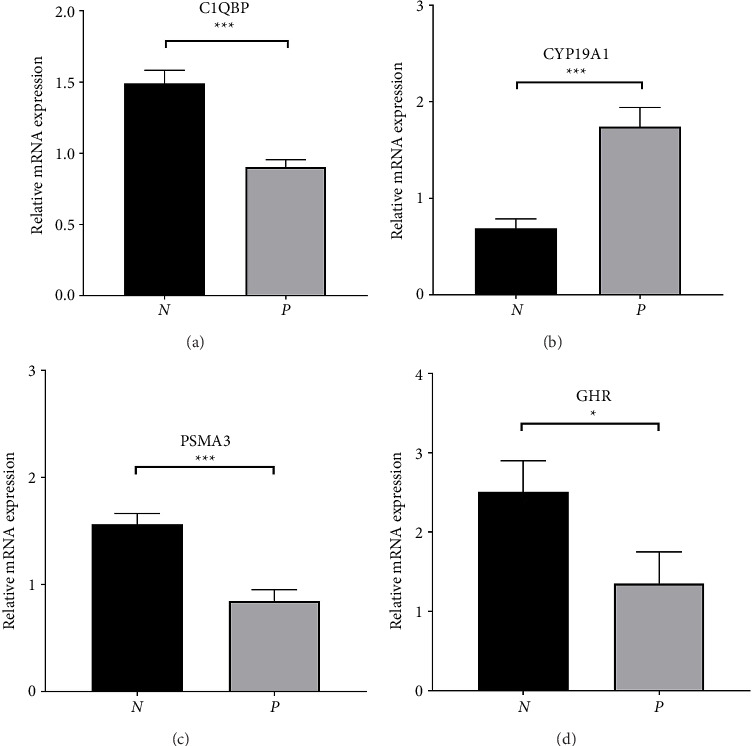
Robust experiment: the mRNA expression of the four hub genes in PE and normal samples. Statistical significance is indicated as follows: ^∗^*p* < 0.05 and ^∗∗∗^*p* < 0.001.

Tissue samples were subjected to qPCR analysis to validate the expression levels of CYP19A1, C1QBP, GHR, and PSMA3. Our qRT-PCR analysis revealed that the expression level of C1QBP, GHR, and PSMA3 were significantly higher in normal tissues compared to PE tissues, whereas the expression levels of CYP19A1 was significantly lower in normal tissues compared to PE tissues. These observations suggest that these genes may play a role in the coagulation and fibrinolysis-related mechanisms of PE (refer to Figure 8(a)). These findings are consistent with the data obtained from bioinformatic analysis.

## 4. Discussion

PE is a pregnancy-specific complication that can cause severe maternal and fetal complications, including severe hypertension, renal failure, disseminated intravascular coagulation, HELLP syndrome, fetal growth restriction, and fetal distress, and it is one of the leading causes of maternal and perinatal mortality [15]. Extensive basic research findings have shown that the core pathological feature of PE is impaired remodeling of the uterine spiral arteries. In PE patients, the remodeling of the uterine spiral arteries is incomplete, as the endothelial cells are not fully replaced by trophoblast cells, and the surrounding smooth muscle remains intact, retaining its contractile function [16]. This results in inadequate dilation of the terminal vessels, severely restricting blood perfusion to the placenta. Endothelial dysfunction leads to changes in osmotic pressure, causing an increase in tissue fluid volume, while simultaneously activating the coagulation cascade and promoting microthrombus formation, further reducing blood volume and creating a vicious cycle that ultimately triggers the development of PE [17, 18]. Therefore, accurate identification of the risk of hypercoagulability during pregnancy is essential as a basis and safeguard for reducing adverse pregnancy outcomes.

This study utilized the microarray data from the premonitory epilepsy GSE10588 dataset, which was obtained from the GEO database. A total of 514 genes related to chromosomes and fibrinolysis were downloaded from the GeneCards database. The PE and normal groups were designated as trait pairs for analysis. WGCNA was applied to analyze the entire gene set of GSE10588 and identify module genes associated with premonitory epilepsy. By intersecting the genes, module genes, and CFRGs, 12 differentially expressed genes (CFR-DEGs) were identified as hub genes. In the training set, the Lasso algorithm and the SVM algorithm were employed to filter the hub gene features. The genes that passed the feature selection of both algorithms were considered characteristic genes, resulting in the identification of 4 biomarkers associated with CFRGs. These biomarkers were recorded as *CYP19A1*, *C1QBP*, *GHR*, and *PSMA3*. These hub genes collectively participate in the cell cycle and proteasome function [19].

The pre-mRNA splicing factor ASF/SF2 is referred to by various alternative names, such as p32, gC1qR, or HABP1 [20]. C1QBP is one of the proteins associated with these names. In addition to its localization in the mitochondrial matrix, the C1QBP protein is distributed across the cell surface, cytoplasm, and nucleus [21, 22]. Functional annotation results exhibited that C1QBP exhibited enrichment in fatty acid metabolism, purine metabolism, and ribosome [23]. It is indicated that membrane-associated ribosomes affected by C1QBP might be involved in the process of mitochondrial-encoded proteins translation and placental mitochondrial dysfunction in PE, and C1QBP perhaps would be indispensable for the gene expression alteration about mitochondrial oxidative phosphorylation and fatty acid oxidation in placentas with PE [24–26].

Hormones are predominantly synthesized by cytochrome P-450 (CYP) enzymes, such as CYP11A1 and CYP19A1, as well as hydroxysteroid dehydrogenases (HSDs), including 3β-HSD and 17β-HSD [27, 28]. Activation of the GHR signaling pathway leads to phosphorylation of signal transducer and activator of transcription 5 (STAT5) in multiple tissues, including the hypothalamus [29]. In pregnancy, several hormones that recruit STAT5, such as prolactin, growth hormone (GH), and placental lactogens, exhibit high secretion levels, where CYP19A1 and GHR signal-related placental hormones might be involved in the pathogenesis of PE [29–31]. Besides, the decrease of ribosome function of NK populations in the preeclamptic placental tissues may be affected by the disorder of *GHR* gene expression [32].

LncRNA PSMA3-AS1 can form RNA duplex structure by binding to pre-PSMA3 to promote PSMA3 transcription. While PSMA3-AS1 exhibits significant involvement in the progression of various types of tumors, its specific mechanisms in the pathogenesis of PE remain unclear. It is noteworthy that the *PSMA3* gene demonstrated enrichment in arachidonic acid metabolism and the notch signaling pathway. The potential of pointing arachidonic derivatives and miRNA involving notch signaling pathway (e.g., miR-34a and miR-214-5) in pregnancy pathologies [33–35], especially PE has been studied, in which the correlation of miR-34a-5p and *C1QBP* were detected in the lncRNA-miRNA-mRNA networks of our study, indicating the potential crosstalk of PSMA3 and C1QBP [36].

When it comes to the regulatory networks targeting the key genes, in addition to the miR-34a-5p mentioned above, the connection of miR-106a-5p and *C1QBP*, miR-106a-5p and *CYP19A1*, miR-19b and *CYP19A1* were displayed. Notably, previous studies have demonstrated the compelling evidence supporting the inhibitory role of c-Myc/miR-17∼92/-106a-363 signaling cascades in trophoblast differentiation, and highly expressed miRNA clusters have been shown to target p21 to suppress hGCM1 and hCYP19A1 [37]. As shown in the disordered expression of *C1QBP* and *CYP19A1* in PE tissues, the dysregulation of this novel signaling pathway may potentially contribute to the pathogenesis of preeclampsia by interfering with the normal induction of trophoblast differentiation.

A study of PE Single-cell profiling demonstrated the biological significance of elevated infiltration of activated NK cells and M2 macrophages in PE. In this study, we observed significant variations in the scores of 14 immune cell types between samples from individuals with preeclampsia and those from normal controls, encompassing macrophages, Type 2 T helper cells, regulatory T cells, and activated dendritic cells, etc. [38, 39]. Furthermore, effector memory CD4/CD8 T cells, CD56dim natural killer cells, activated dendritic cell exhibited the strongest negative correlations with four hub genes (both correlations larger than 0.3, *p* < 0.05), which were consistent with previous findings and further detailed potential NK cell subtypes. Research indicates that the multifunctional partner protein p32/C1qbp, playing a role in the mitochondrial matrix, supports mitochondrial metabolism and dendritic cell maturation. In experiments related to GHR knockout mice, Stout et al. demonstrated a decrease in gene expression levels in cells derived from monocytes (dendritic cells and macrophages). Additionally, Ishikawa et al. found an increase in marker gene expression for natural killer/natural killer T cells.

We predicted potential therapeutic drugs for 61 biomarkers, and the biomarker-drug network comprises relationships such as CYP19A1-Tamoxifen, GHR-Pegvisomant, HA-C1QBP, and CYP19A1-Paclitaxel. There is existing evidence supporting the transplacental transfer and distribution of pravastatin during pregnancy and the inhibitory effects of which on the ATP-dependent uptake of Paclitaxel [40], on the other hand, the transcription factors STOX2 which was associated with fetal growth restricted PE could enhance paclitaxel resistance. These findings exhibited a potential of Paclitaxel in PE that is worth exploring. Moreover, tamoxifen treatment can conditionally induce the downregulation of pleiotrophin (PTN), and PTN downregulation increases the risk of PE after the transplantation of cryopreserved embryos [41]. Pegvisomant is commonly used in clinical settings to treat acromegaly in women, and PE may be a common and significant complication of acromegaly [42]. Numerous studies have investigated the pharmacokinetic interactions and pathological regulatory functions of HA in PE [43–46].

Nevertheless, it is crucial to recognize the limitations inherent in our study. Firstly, our bioinformatics analysis was based solely on target data obtained from the publicly available GEO database, and the utilization of bioinformatic algorithms may introduce inherent limitations and biases. Moreover, although we identified 12 hub genes as potential biomarkers associated with PE coagulation and fibrinolysis, it is essential to conduct larger-scale, multicenter, prospective clinical cohort studies to evaluate the clinical applicability and effectiveness of our findings. Likewise, the functional annotations involving the hub genes from bioinformatics results remain to be confirmed by combining the additional functional experimental data from in vitro or animal models with the comprehensive drug sensitivity analysis and single-cell clustering analysis focusing on the correlation between differential immune cells and hub genes. Additionally, our study focused exclusively on protein-coding genes, yet emerging evidence indicates that noncoding RNAs, including lncRNAs and microRNAs, play a significant role in the pathogenesis of PE. Therefore, future investigations should also consider the involvement of these noncoding RNAs to achieve a comprehensive understanding of the molecular mechanisms underlying PE. We aim to address these limitations in our future research endeavors.

## 5. Conclusion

In conclusion, our WGCNA-based study has identified CYP19A1, C1QBP, GHR, and PSMA3 as potential mediators involved in the progression of PE, particularly in relation to coagulation and fibrinolysis processes. These gene findings hold promising implications as diagnostic biomarkers and therapeutic targets for PE, offering potential advancements in the diagnosis and treatment of this condition. In order to obtain a comprehensive insight into the pathogenesis of preeclampsia, additional prospective studies of larger scale are warranted. We will continue to closely monitor the advancements in research concerning biomarkers associated with coagulation and fibrinolysis and their relevance to PE.

## Data Availability

The provision of data will be in accordance with the specified requirements.
